# Comparative Whole Genome Analysis and Targeted Validation of Variants in Three Greek Indigenous Sheep Breeds

**DOI:** 10.3390/cimb48050480

**Published:** 2026-05-05

**Authors:** Maria-Anna Kyrgiafini, Georgios Stamatellos, Costas Stamatis, Zissis Mamuris

**Affiliations:** Laboratory of Genetics, Comparative and Evolutionary Biology, Department of Biochemistry and Biotechnology, University of Thessaly, 41500 Larissa, Greece; mkyrgiafini@uth.gr (M.-A.K.); stamatellosgeo@gmail.com (G.S.); kstamatis@bio.uth.gr (C.S.)

**Keywords:** indigenous breeds, *Ovis aries*, whole genome sequencing, genetic diversity

## Abstract

Indigenous sheep breeds represent valuable reservoirs of genetic diversity shaped by long-term adaptation to local environments and management systems. Greek autochthonous sheep breeds remain underrepresented in genomic and functional studies. The objective of this study was to characterize and compare coding sequence variation in three indigenous Greek sheep breeds—Lesvos (LES), Serres (SER), and Thrace (THR)—and to identify shared and breed-associated functional patterns. The study was designed using a two-stage approach, comprising a discovery (exploratory) phase and a validation phase. In the discovery phase, whole genome sequencing data (one animal per breed; total *n* = 3; mean sequencing depth ~36.9×) were analyzed to identify protein-altering exonic variants, focusing on missense single-nucleotide polymorphisms (SNPs) and exonic insertions/deletions (indels). Variants were examined at breed-specific and comparative levels, followed by functional enrichment analyses using Gene Ontology (GO) and KEGG pathways. Normalized variant density metrics identified genes with elevated polymorphism levels. In the validation phase, a subset of prioritized missense SNPs was genotyped in an independent cohort of 54 animals (18 per breed) using MassARRAY genotyping. Genes harboring prioritized missense SNPs showed a conserved enrichment profile across breeds, dominated by genome maintenance, DNA repair, cytoskeletal organization, and core regulatory functions. Distinct breed-associated patterns were also observed. LES showed enrichment in metabolic, biosynthetic, and sensory-related processes, SER in regulatory and signaling functions, and THR in cytoskeletal, extracellular matrix, and organelle-associated pathways. Polymorphism density analyses highlighted highly variable genes across breeds, including olfactory receptor (OR) gene families, keratin-associated protein genes (*KRTAPs*), and loci involved in immune and regulatory functions (e.g., *PRKDC*, *CDH15*). The validation phase confirmed the expected allele frequency patterns for most prioritized SNPs, supporting the robustness of the approach. This study identifies functionally relevant coding variation across Greek indigenous sheep breeds, revealing conserved genomic patterns and breed-associated signatures linked to metabolic, structural, and regulatory processes.

## 1. Introduction

Livestock plays a pivotal role in global agriculture, providing nutrition, income, and cultural significance to millions of people worldwide [1,2]. Within this context, sheep (*Ovis aries*) are particularly notable for their adaptability to diverse environments and their production of multiple products, including meat, milk, and wool. Their ability to thrive in marginal areas renders them especially valuable in Mediterranean and semi-arid regions, where they contribute not only to food production but also to the preservation of rural heritage and landscape management [3].

Livestock farming is one of the most widespread agricultural practices in Greece, with small ruminants, especially sheep and goats, at its core. Specifically, Greece has the third-largest sheep flock in the European Union [4]. Over centuries, more than 20 indigenous sheep breeds have developed in Greece due to geographical isolation, transhumance, natural and human-driven selection, crossbreeding, and genetic drift [5]. These autochthonous breeds are reared primarily for milk production, supporting a vibrant dairy sector that produces numerous Protected Designation of Origin (PDO) and Protected Geographical Indication (PGI) products, while meat plays a secondary role. Phenotypically distinct and well-adapted to heterogeneous Mediterranean environments, local Greek breeds represent a valuable pool of genetic diversity shaped by centuries of adaptation to the Greek environment [6]. These breeds sustain traditional livelihoods and regional economies while exhibiting adaptive traits such as heat stress tolerance, efficiency in extensive grazing systems, and resilience to endemic diseases. Such attributes are increasingly critical in the context of climate change and landscape transformations [7]. However, many indigenous Greek breeds are experiencing demographic decline and genetic erosion, primarily as a result of production intensification and indiscriminate crossbreeding. Therefore, conserving these genetic resources is crucial for maintaining biodiversity and ensuring the long-term sustainability of animal production systems in Greece [6,7].

During the past two decades, the application of molecular tools has transformed the study of livestock diversity and adaptation. Early approaches, which relied on microsatellites and mitochondrial DNA, provided valuable insights into phylogeny and population structure, but were limited in scope [8,9]. The development of high-density SNP arrays accelerated genomic studies in sheep, enabling the characterization of breed relationships, selection signatures, and inbreeding levels across global populations [9,10]. However, single-platform genotyping captures only a fraction of genetic variation, whereas whole genome sequencing (WGS) offers a more comprehensive view, including rare variants and structural polymorphisms with potential functional significance. WGS has already been employed to investigate domestication history, identify genomic regions under selection, and reveal adaptive traits in sheep from diverse environments [11], making WGS an essential tool for both evolutionary research and the design of sustainable breeding programs.

Despite Greece’s rich pool of indigenous sheep, genomic resources for these populations remain scarce compared to cosmopolitan breeds, leading to significant knowledge gaps in our understanding of their evolutionary history and adaptive potential. Recent efforts have begun to address these long-standing gaps in Greek sheep, most notably through the first WGS dataset involving several breeds [12]. However, substantial gaps still exist for many regionally important breeds, and comparative WGS across geographically and ecologically diverse Greek populations remains scarce [6]. These limitations hinder the scientific exploration of adaptive mechanisms and the practical application of genomic data to breed conservation, tracing animal origins, and implementing breeding programs tailored to local environmental conditions. Although expanding WGS sampling of insular and mainland Greek sheep breeds is critical to capture the full spectrum of genetic diversity, equally essential is the comparative analysis of genomic variants between these breeds to uncover both shared and unique adaptive mechanisms. Addressing this deficit is particularly urgent given the dual challenges of climate change and ongoing genetic erosion, which threaten not only the sustainability of Greek sheep farming but also the broader resilience of Mediterranean small ruminant production systems [6,13].

**Figure 1:**
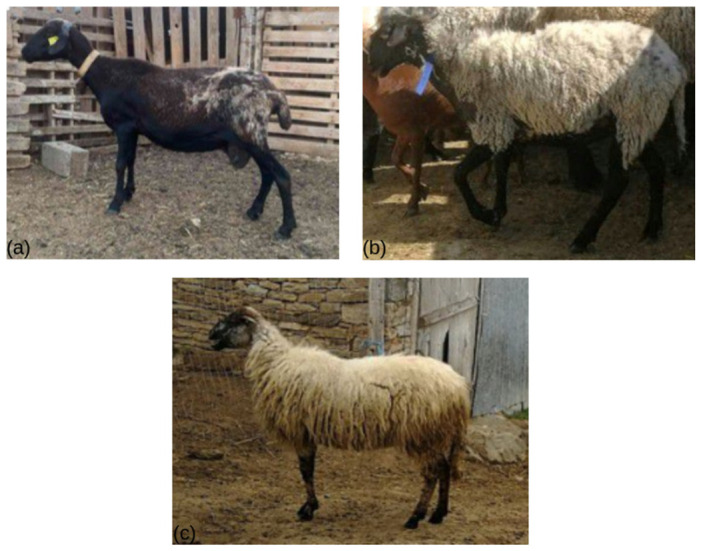
Representative individuals of the three Greek indigenous sheep breeds included in this study: (**a**) Lesvos (LES), (**b**) Serres (SER), and (**c**) Thrace (THR).

**Figure 2:**
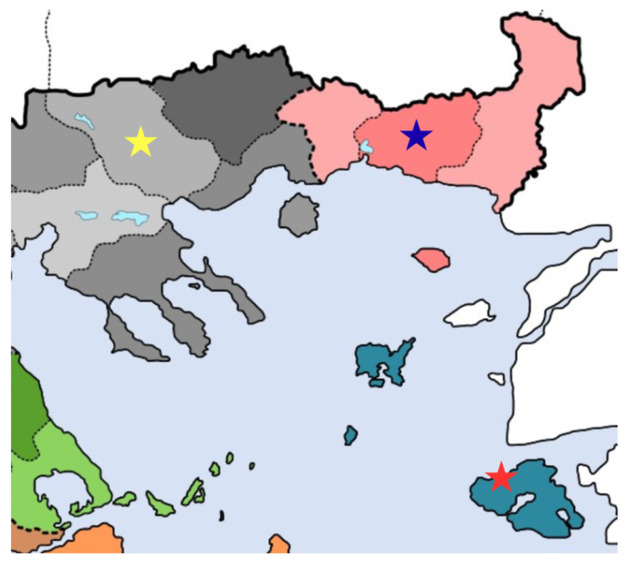
Geographic distribution of the three indigenous sheep populations examined in this study. Sampling regions are indicated by colored stars: LES (red), SER (yellow), and THR (blue).

In this study, we investigate three Greek autochthonous sheep breeds—Lesvos (LES), Thrace (THR), and Serres (SER)—each representing distinct ecological and management contexts. The three indigenous breeds represent distinct geographical regions and production systems within Greece. The LES breed, originating from the island of Lesvos, is typically characterized by a white coat with dark pigmentation on the nose and limbs, long semi-fat tails, and pendulous ears. In contrast, the SER breed, reared mainly in northern mainland Greece, exhibits a more robust body size and is characterized by a predominantly white coat with dark pigmentation in the head, limbs, and ventral body region. Animals display a relatively narrow head with a slightly convex nasal profile and large, horizontally oriented ears, while rams often carry well-developed horns. The THR breed, also native to northern Greece, is generally smaller in size, with narrower ears, a straight nasal profile, and long, thin tails. Representative individuals of each breed are shown in Figure 1, while their geographical distribution is illustrated in Figure 2.

In terms of population size and production characteristics, the LES breed represents one of the most widespread indigenous Greek sheep populations, with an estimated population of approximately 260,000 animals. This medium-sized breed is primarily oriented towards milk production, with average yields ranging between 140 and 160 kg per lactation, and is well known for its adaptability to harsh environmental conditions and its ability to efficiently utilize low-quality pastures. A large proportion of the milk produced is traditionally used for cheese production, highlighting the economic importance of the breed in local agro-pastoral systems. Adult body weight typically reaches approximately 65–70 kg in males and 45–55 kg in females [14].

The THR breed represents a rare genetic resource, with fewer than 1000 purebred individuals, is primarily reared by Pomak communities in the mountainous areas of northwestern Rodopi under extensive production systems. This small-sized breed is highly adapted to harsh environmental conditions and efficiently exploits marginal grazing resources. Adult body weight typically ranges between 45 and 55 kg in males and 35–45 kg in females, while height at withers is approximately 62–65 cm and 55–60 cm for males and females, respectively. The breed produces coarse but good-quality wool and is primarily used for milk and meat production under low-input systems. Milk yield ranges between 60 and 100 kg per lactation, while meat production is relatively low [15].

Finally, the SER breed represents a relatively small but important population, with approximately 6000 animals. It is a large-bodied breed, well-suited for semi-intensive and extensive production systems, and is valued for both milk and meat production. Adult body weight typically reaches approximately 80–90 kg in males and 55–65 kg in females. In terms of productive performance, lambs reach weights of approximately 14–17 kg at weaning (45–60 days), while the average milk yield is estimated at around 130–140 kg per lactation, with a production period of approximately 200–220 days. The breed is well adapted to mountainous and semi-mountainous environments, efficiently utilizing available grazing resources [14].

Building on a whole genome sequencing (WGS) dataset, our objective was to investigate genome-wide variation in the above Greek breeds (LES, SER, THR), with a particular focus on missense variants, given their potential to alter protein function and contribute to breed-specific adaptive traits. To explore the biological relevance of these variants, we conducted functional annotation and enrichment analyses through Gene Ontology (GO) and Kyoto Encyclopedia of Genes and Genomes (KEGG) frameworks. Furthermore, a subset of prioritized missense SNPs was validated in an independent cohort of animals using MassARRAY genotyping to assess their allele frequency distribution across breeds. By integrating genomic discovery with population-level validation, this study provides new insights into the adaptive genomic landscape of indigenous Greek sheep and strengthens the foundations for their conservation and sustainable management.

## 2. Materials and Methods

### 2.1. Study Design Overview

The present study was designed to investigate genomic variation across three indigenous Greek sheep breeds (LES, SER, THR) using a two-stage approach consisting of a discovery phase and a validation phase. In the discovery phase, whole genome sequencing (WGS) data from a representative set of animals (one individual per breed; total *n* = 3), obtained from a previously published dataset [12], were used to identify and filter SNPs and insertions/deletions (indels), and to perform comparative genomic analyses between the three populations. In the validation phase, an independent cohort of animals (*n* = 54; 18 per breed), consisting of samples collected and processed by our team, was used to evaluate the population-level distribution of the selected variants. Specifically, a panel of missense SNPs identified during the discovery phase was genotyped using a MassARRAY-based approach.

**Figure 3:**
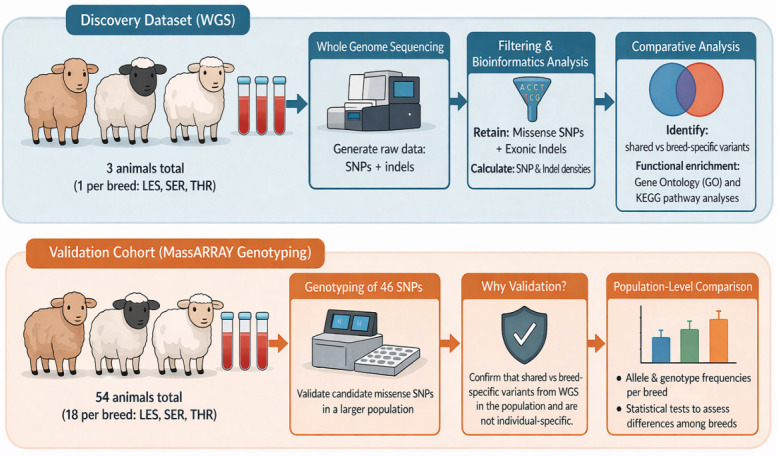
Schematic overview of the study design and analytical workflow. The analysis was performed in two stages: (i) a discovery phase, where whole genome sequencing data from three representative animals (one per breed: LES, SER, THR) were used for variant identification and filtering, and (ii) a validation phase, where a panel of selected missense SNPs was genotyped in an independent cohort of 54 animals (18 per breed) using a MassARRAY-based approach to assess their distribution at the population level.

The animals included in the discovery and validation phases were independent and did not overlap. This two-tier strategy enabled both the identification of candidate variants at the genome-wide level and their subsequent assessment in a larger, breed-representative population. A schematic overview of the study workflow is provided in Figure 3.

### 2.2. Discovery Dataset: Biological Material & Whole Genome Sequencing

For this study, we utilized WGS data originally produced by Tsoureki et al. (2025) [12]. Specifically, blood samples were collected by Tsoureki et al. (2025) [12] from three different animals representing three indigenous Greek sheep breeds: “Lesvos” (LES), “Serres” (SER), and “Thrace” (THR). The animals were selected based on their morphological characteristics and geographic origin to ensure purebred representation. Sampling was performed via jugular venipuncture, and the collected blood was transferred into EDTA-containing tubes and stored at −20 °C. Genomic DNA was extracted using the NucleoSpin Blood QuickPure kit (MACHEREY-NAGEL, Düren, Germany), following the manufacturer’s instructions. DNA concentration was measured with a Qubit 4 Fluorometer using the Qubit dsDNA BR Assay Kit (Thermo Fisher Scientific, Waltham, MA, USA), and DNA integrity was assessed by agarose gel electrophoresis [12].

Library preparation and WGS were also conducted by Tsoureki et al. (2025) [12]. In brief, whole genome libraries were constructed using the Illumina Nextera DNA Flex kit (Illumina Inc., San Diego, CA, USA), following the manufacturer’s instructions. The libraries were purified with AMPure XP Beads (Beckman Coulter, Brea, CA, USA), and their concentrations were measured using a Qubit 4 Fluorometer and the Qubit dsDNA BR Assay Kit (Thermo Fisher Scientific, Waltham, MA, USA). Library size and quality were assessed with the 5200 Fragment Analyzer system (Agilent Technologies Inc., Santa Clara, CA, USA), while quantification of the libraries was performed on a Rotor-Gene Q real-time PCR system (Qiagen, Hilden, Germany) using the KAPA Library Quantification kit (KAPA BIOSYSTEMS, Wilmington, MA, USA). Sequencing was conducted on a NovaSeq 6000 platform (Illumina Inc., San Diego, CA, USA) [12].

### 2.3. Bioinformatics Analysis

For the present study, raw sequencing data from three Greek sheep breeds (LES, THR, SER) were retrieved from the publicly available dataset of Tsoureki et al. (2025) [12]. Data were obtained from the NCBI Sequence Read Archive (SRA) under BioProject accession number PRJNA1246525 and were used for comparative whole genome analyses. All downstream bioinformatics analyses, including variant calling, filtering, annotation, and variant prioritization, were performed independently by our team in the context of this study.

Raw FASTQ files were subjected to quality assessment using FastQC [16]. Adapter sequences and low-quality bases (PHRED < 30) were trimmed with Trimmomatic (v0.39) [17]. The resulting high-quality reads were aligned to the *Ovis aries* reference genome (Oar_rambouillet_v1.0), obtained from the Ensembl database [18], using the Burrows–Wheeler Aligner (BWA) (version 0.7.17) [19]. PCR duplicates were identified and removed using Picard tools (http://broadinstitute.github.io/picard/, accessed on 9 March 2026) before further analysis. Alignment files in SAM format were subsequently converted to sorted BAM files with SAMtools (v1.19.2) [20]. Variant calling, including both single-nucleotide polymorphisms (SNPs) and insertions/deletions (indels), was performed using SAMtools (v1.19.2) [20]. Raw variant sets were filtered based on the following criteria: (1) Read depth (DP) at the variant position > 4; (2) Mapping quality (MQ) > 20. Variant call format (VCF) files were generated for each sample (LES, THR, SER). The resulting high-confidence variants were functionally annotated using ANNOVAR (v20191024) [21], which provided information on their genomic context (e.g., exonic, intronic, intergenic), predicted effects on protein-coding regions, overlaps with known gene annotations, etc.

### 2.4. Investigation of Variants & Comparative Analysis

In this study, we aimed to investigate genetic variants across three Greek sheep breeds to gain preliminary insights into shared and unique variations, as well as to explore variants that may contribute to breed-associated adaptive traits. To achieve this, we employed an approach that prioritized missense variants and insertions/deletions (indels) in each breed due to their potential to alter protein function and contribute to phenotypic diversity. Subsequently, we performed gene ontology (GO) and KEGG pathway analyses to evaluate the potential biological impact of these variants. Additionally, we identified genomic regions with high SNP or indel density, as these regions may indicate signatures of selection, increased mutation rates, or functionally important loci involved in breed-specific adaptations. Finally, we conducted a comparative analysis to examine the genetic similarities and differences among the three breeds.

Specifically, to facilitate downstream analyses, two annotated variant datasets were generated for each breed: one containing exonic single-nucleotide polymorphisms (SNPs) and the other containing exonic insertions and deletions (indels). Each dataset included key variant information, such as the variant ID (rsID), chromosome (CHR), genomic position (POS), reference (REF), and alternate (ALT) alleles, as well as the corresponding gene annotation. Initial quality control involved the removal of duplicate entries and variants with missing or invalid gene annotations. For SNPs, only missense variants were prioritized, as they are more likely to directly impact protein structure or function. In contrast, all exonic indels were retained for further analysis, due to their high potential to disrupt coding sequences.

For each breed, genes harboring missense SNPs and exonic indels were retrieved and analyzed separately, resulting in six gene lists in total (two per breed). These lists were submitted to g:Profiler [22] for exploratory functional enrichment analysis, using *Ovis aries* as the background organism. Multiple testing correction was performed using the g:SCS (Set Counts and Sizes) method, with a significance threshold of *p* < 0.05. Analyses were conducted under the “all known genes” statistical domain. Functional categories included Gene Ontology (GO) terms (Biological Process, Molecular Function, and Cellular Component) [23,24], as well as KEGG pathways [25], providing an overview of the potential biological roles of the identified genes.

Subsequently, variant counts were calculated separately for SNPs and indels to generate “SNP Count per Gene” and “Indel Count per Gene” for each breed. Gene coordinates were retrieved from Ensembl BioMart [26] and used to calculate gene lengths in kilobases (kb). Normalized variant densities were then computed as SNPs per kilobase (SNPs/kb) and indels per kilobase (indels/kb). These normalized metrics enabled the identification of genes with elevated polymorphism density.

Pairwise comparisons (LES-THR, LES-SER, THR-SER), as well as a three-way comparison among all breeds, were performed to identify shared and breed-specific genes and SNPs. The three-way analysis enabled the detection of genes and variants common to all breeds, as well as those unique to each breed. Identifying both common (which may reflect conserved or essential functions) and breed-specific variants (which may underlie local adaptation or distinct phenotypic traits) provides valuable insights into the genetic basis of diversity among Greek sheep populations. In total, the gene-level and SNP-level comparisons yielded thirteen distinct lists each, representing shared and unique elements derived from both pairwise and three-way comparisons. Finally, each gene list was submitted to g:Profiler [22] for functional enrichment analysis, following the same parameters used in the initial breed-specific analyses, described earlier. This step aimed to uncover biological processes and pathways uniquely or commonly affected across breeds, offering further preliminary insight into potential shared functions or breed-specific adaptations.

All analyses were performed using custom Python scripts in a Python 3.12 environment, primarily utilizing the Pandas library (v2.2.2).

### 2.5. Selection of Candidate Missense Variants for Validation

To assess whether putatively breed-specific coding variants identified in the WGS comparative analysis segregate at the population level, a subset of missense SNPs was selected for experimental validation.

Candidate variants were selected from the pool of missense SNPs classified as unique to one breed in the comparative analysis. Priority was given to high-confidence variants that passed quality filtering criteria in the WGS dataset and were located within well-annotated protein-coding genes. When multiple variants were present within the same gene or genomic region, representative SNPs were chosen to minimize redundancy and ensure broader genomic coverage. Biological relevance was also considered during prioritization, particularly for genes implicated in metabolic processes, immune function, sensory perception, skin and wool biology, or other pathways highlighted in functional enrichment analyses. Additionally, variants were screened for technical suitability for MassARRAY assay design, including the absence of nearby polymorphisms that could interfere with primer binding and genotyping accuracy. Based on these criteria, a panel of 46 missense SNPs was selected for subsequent analysis and genotyping.

### 2.6. Validation Cohort: Animals and DNA Samples

To evaluate the population-level distribution of the candidate missense variants identified in the WGS analysis, an independent validation cohort of 54 animals was assembled. This cohort included 18 individuals from each of the three Greek indigenous sheep breeds (LES, THR, SER). Animals were selected to represent unrelated individuals, as far as could be determined from available farm records, to minimize potential bias due to close kinship. Animal handling and experimental procedures adhered to European regulations on animal welfare. Since all procedures were non-invasive, no specific license was required.

For the analysis, blood samples were collected in EDTA tubes, transported on ice, and stored at −20 °C until DNA extraction. Genomic DNA was isolated using the PureLink Genomic DNA Mini Kit(Invitrogen, Waltham, MA, USA—Catalog number: K182002), following the manufacturer’s instructions. DNA concentration was assessed using a Qubit 4 Fluorometer Thermo Fisher Scientific, Waltham, MA, USA) and the Qubit dsDNA BR Assay Kit (Thermo Fisher Scientific, Waltham, MA, USA), while integrity was evaluated through agarose gel electrophoresis. Only samples meeting quality requirements were included in the downstream genotyping analyses.

### 2.7. MassARRAY Assay Design and Genotyping

Genotyping of the 46 selected missense SNPs was performed using the Agena Bioscience MassARRAY^®^ system (Agena Bioscience, San Diego, CA, USA), which is based on matrix-assisted laser desorption/ionization time-of-flight (MALDI-TOF) mass spectrometry. The MassARRAY assays were designed and executed by Inqaba Biotec™ (Inqaba Biotechnical Industries, Pretoria, South Africa), following standard iPLEX genotyping protocols. Briefly, genomic regions flanking each selected SNP were used for primer design to enable multiplex PCR amplification. During assay development, the sequence context was evaluated to minimize potential interference from nearby polymorphisms and to ensure optimal amplification and extension efficiency. Genotyping involved multiplex PCR amplification of target regions, enzymatic cleanup using shrimp alkaline phosphatase (SAP), and a single-base extension reaction (iPLEX) with mass-modified terminator nucleotides. The resulting extension products were dispensed onto a SpectroCHIP array and analyzed via MALDI-TOF mass spectrometry for allele discrimination [27].

Raw spectral data were processed using MassARRAY Typer software 4.1 (Agena Bioscience) to assign genotypes based on automated clustering algorithms. Genotype calls were subsequently assessed for quality and consistency prior to downstream statistical analyses.

### 2.8. Genotyping Quality Control & Statistical Analyses

Genotype calls were subjected to quality control prior to downstream analyses. SNP-level and sample-level call rates were assessed to ensure data reliability. Variants or samples with low call rates (threshold set at ≥90% completeness) were excluded from further analysis. The quality of genotype clustering was visually inspected using the MassARRAY Typer output to confirm clear allele discrimination.

Allele and genotype frequencies were calculated separately for each breed (LES, SER, THR). To evaluate differences in allele distribution among breeds, global comparisons were performed using chi-square tests or Fisher’s exact tests where appropriate. Pairwise breed comparisons were also conducted to further assess differentiation between specific breed pairs. To account for multiple hypothesis testing across the 46 SNPs, *p*-values were adjusted using the Benjamini–Hochberg false discovery rate (FDR) correction method. Statistical significance was defined at a corrected q-value < 0.05.

All statistical analyses were performed in R version 4.3.2. using standard packages for contingency testing and data handling.

## 3. Results

### 3.1. *Discovery Dataset*

#### 3.1.1. Variant Discovery & Prioritization per Breed

Whole genome variant discovery was performed independently for each breed included in the study: Lesvos (LES), Thrace (THR), and Serres (SER), identifying a total of 81,857 SNPs in LES, 78,783 SNPs in SER, and 79,312 SNPs in THR. In addition, 1996 (LES), 1925 (SER), and 2003 (THR) indels were identified.

To provide an overview of the functional distribution of SNPs, variants were further categorized based on their predicted effects. Across the three breeds, the majority of SNPs corresponded to synonymous and nonsynonymous variants, with smaller proportions of stop-gain, stop-loss, and variants of unknown effect (Table 1). To focus on variants with potential functional relevance, a stepwise filtering approach was applied. First, SNPs were filtered to retain nonsynonymous variants, which are more likely to affect protein function. Subsequently, the variants were further restricted to those annotated within protein-coding genes. Across the three breeds, a total of 19,120 missense SNPs were detected in LES, 18,513 in SER, and 18,459 in THR. Using the same variant-calling and filtering workflow, indel discovery identified 1015 exonic indels in LES, 983 in SER, and 1016 in THR.

**Figure 4:**
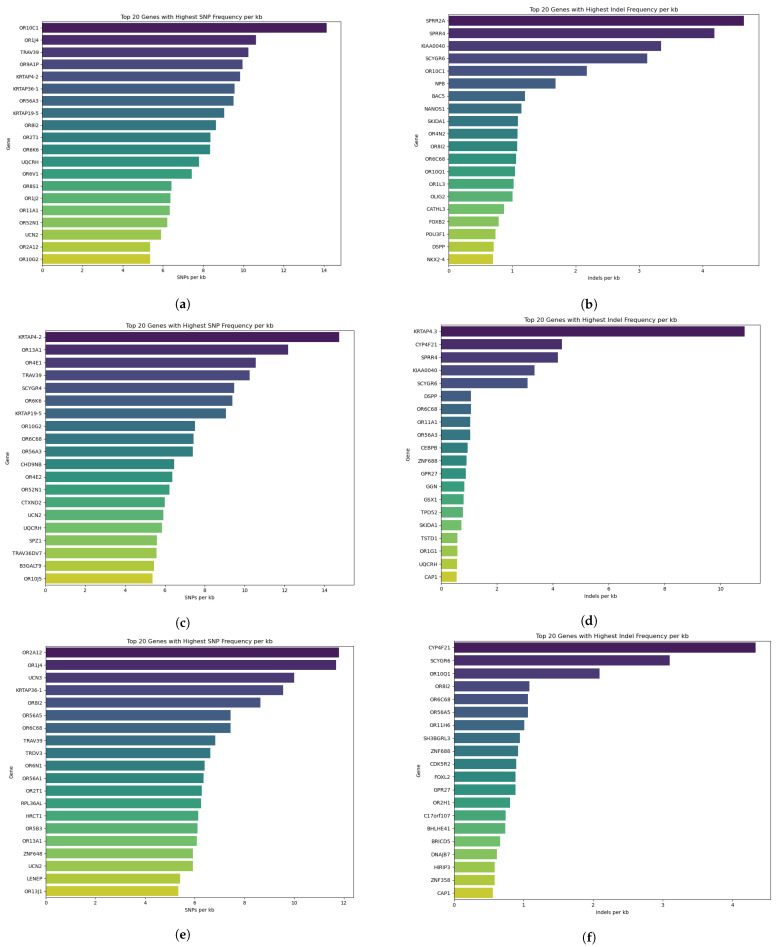
Genes exhibiting the highest normalized variant density across the three Greek sheep breeds. Normalized variant densities, expressed as single-nucleotide polymorphisms per kilobase (SNPs/kb) and insertions/deletions per kilobase (indels/kb), were calculated for all genes to account for differences in gene length. Figures show the genes with the highest SNP density in LES (**a**), highest indel density in LES (**b**), highest SNP density in SER (**c**), highest indel density in SER (**d**), highest SNP density in THR (**e**), and highest indel density in THR (**f**). Only the top 20 genes per category are displayed.

To examine the distribution of coding variants throughout the genome, missense SNPs and exonic indels were mapped to their corresponding protein-coding genes for each breed. Normalized variant density metrics, expressed as single-nucleotide polymorphisms per kilobase (SNPs/kb) and insertions/deletions per kilobase (indels/kb), were calculated for all genes in each breed. This approach accounted for differences in gene length and enabled the identification of genes with disproportionately high levels of coding sequence polymorphism. Based on these metrics, the genes with the highest SNP densities were *OR10C1*, *OR1J4*, *TRAV39*, *OR9A1P*, and *KRTAP4-2* in LES (Figure 4a); *KRTAP4-2*, *OR13A1*, *OR4E1*, *TRAV39*, and *SCYGR4* in SER (Figure 4c); and *OR2A12*, *OR1J4*, *UCN3*, *KRTAP36-1*, and *OR8I2* in THR (Figure 4e). Similarly, the genes displaying the highest indel densities were *SPRR2A*, *SPRR4*, *KIAA0040*, *SCYGR6*, and *OR10C1* in LES (Figure 4b); *KRTAP4-3*, *CYP4F21*, *SPRR4*, *KIAA0040* and *SCYGR6* in SER (Figure 4d); and *CYP4F21*, *SCYGR6*, *OR10Q1*, *OR8I2* and *OR6C68* in THR (Figure 4f).

To facilitate the interpretation of the identified genes, the top-ranked genes based on normalized SNP and indel densities are summarized in Supplementary Table S1a–f, including their corresponding Ensembl Gene IDs and gene descriptions.

#### 3.1.2. Functional Enrichment Analysis per Breed

To examine the potential biological significance of genes with coding sequence variation, a functional enrichment analysis was conducted independently for each breed, utilizing gene sets derived from prioritized missense SNPs and exonic indels. Gene Ontology (GO) categories, including Biological Process (BP), Molecular Function (MF), and Cellular Component (CC), as well as KEGG pathways, were assessed to identify functional categories linked to variant-containing genes within each breed.

Analysis of genes carrying prioritized missense SNPs revealed a core set of enriched biological processes shared between all three breeds, predominantly related to genome maintenance and DNA repair, cilium assembly and organization, and cytoskeletal and cell projection–related processes. At the molecular function level, common enrichment patterns included ion channel and calcium signaling activity, regulation of small GTPase and nucleoside triphosphatase activity, and nucleic acid–associated and ATP-dependent catalytic activity, indicating a conserved functional backbone linked to cellular structure, signaling, and genome integrity. KEGG pathway analysis further supported this shared profile, with recurrent enrichment in genome maintenance and protein biosynthesis pathways, cytoskeletal and muscle-related pathways, and ECM–receptor interaction (Table 2).

Despite this shared functional core, breed-specific enrichment profiles were evident. In LES, missense SNP–associated genes showed additional enrichment in cofactor and metabolic processes, including pathways related to vitamin digestion and absorption, as well as specialized biological processes such as photoreceptor cell maintenance. At the molecular function level, LES-specific enrichment included chromatin-associated binding and epigenetic regulation, glycosyltransferase and carbohydrate-modifying activity, ABC transporter activity, and functions related to vesicular trafficking and small GTPase signaling. KEGG analysis further highlighted ABC transporter pathways and nutrient-related metabolic pathways (Table 2).

In SER, breed-specific enrichment for missense SNP–harboring genes was mainly associated with structural and cell-cycle-related processes, including genome maintenance coupled with cell cycle regulation, microtubule-based cytoskeletal organization, and cell projection assembly. Molecular function enrichment in SER emphasized ion channel and calcium signaling activity, regulation of small GTPases, nucleic acid–associated catalytic activity, and lipid transport and lipid metabolic functions, together with carbon–oxygen lyase activity. KEGG pathway enrichment in SER was dominated by DNA repair–related pathways, cytoskeletal and motor protein pathways, and ECM–receptor interaction (Table 2).

In THR, missense SNP–associated genes were enriched for biological processes related to genome maintenance and DNA repair, cilium assembly and organization, microtubule-based cytoskeletal processes, cell projection assembly, and extracellular structure organization. At the molecular function level, THR-specific enrichment included nucleic acid–associated and ATP-dependent catalytic activity, regulation of small GTPase signaling, ion channel activity, and lipid metabolic and ester hydrolysis functions. KEGG pathway analysis additionally identified enrichment in metabolic and organelle-associated pathways, including lysine degradation and peroxisome, alongside ABC transporter pathways, cytoskeletal and muscle-related pathways, and ECM–receptor interaction (Table 2).

Functional enrichment analysis of genes harboring prioritized exonic indels revealed a more homogeneous pattern across breeds, with dominant enrichment in developmental and morphogenetic processes, organelle organization, and cellular responses to stress or external stimuli. In LES, indel-associated genes were additionally linked to the regulation of metabolic processes, whereas SER and THR showed consistent enrichment in the regulation of RNA metabolism and gene expression. At the molecular function level, indel-associated genes across all breeds were primarily enriched for general catalytic activities, including hydrolase and transferase functions, as well as enzyme binding and regulatory activities, nucleoside phosphate binding, and ion binding, with guanyl nucleotide exchange factor activity specifically enriched in THR (Table 3).

The most significantly enriched GO terms and KEGG pathways identified for each breed are summarized in Table 2 and Table 3 for prioritized SNPs and indels, respectively.

### 3.2. Comparative Analysis

To directly assess genetic similarities and differences between the three Greek sheep breeds, comparative analyses were conducted at both the variant and gene levels. Pairwise comparisons between the breeds (Lesvos–Thrace, Lesvos–Serres, and Thrace–Serres), along with a three-way comparison that included all populations, were used to identify shared and breed-specific missense SNPs and variant-associated genes. The three-way analysis allowed for the delineation of a core set of variants and genes common to all breeds, as well as population-specific components of coding sequence variation. To further evaluate the biological relevance of these shared and unique gene sets, functional enrichment analysis based on Gene Ontology (GO) terms and KEGG pathways was subsequently performed.

#### 3.2.1. SNP-Level Comparative Analysis

Pairwise and three-way comparisons of the SNP datasets were conducted to assess shared and breed-specific nucleotide variation between LES, SER, and THR. Pairwise intersections revealed substantial overlap between breeds: 11,046 SNPs were shared between LES and SER, 11,098 SNPs between LES and THR, and 10,827 SNPs between SER and THR. Each comparison also identified a significant number of SNPs unique to individual breeds, with 8074 SNPs exclusive to LES and 7467 to SER in the LES–SER comparison; 8022 SNPs exclusive to LES and 7361 to THR in the LES–THR comparison; and 7686 SNPs unique to SER and 7632 to THR in the SER–THR comparison.

**Figure 5:**
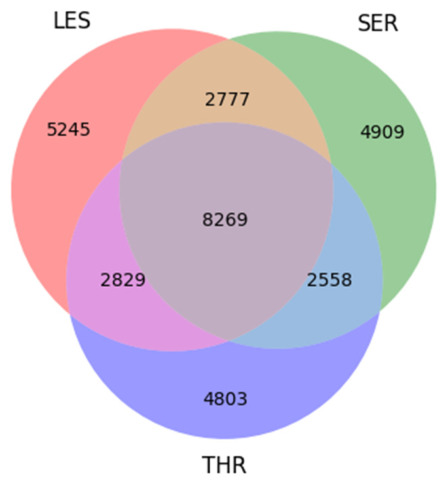
Venn diagram illustrating shared and breed-specific missense SNPs among three Greek sheep breeds (LES, SER, THR).

The three-way intersection identified 8269 SNPs common to all three breeds, representing a shared core of coding variation. Breed-exclusive SNP sets included 5245 SNPs in LES, 4909 in SER, and 4803 in THR, indicating that despite a common genetic backbone, each breed retains a notable pool of unique variants. The results are illustrated in a Venn diagram (Figure 5).

It should be highlighted that this analysis provides a descriptive overview of shared and breed-specific variation at the SNP level based on whole genome sequencing data from the discovery dataset. A more detailed and statistically supported evaluation of allele frequency differences and variant differentiation across breeds is subsequently performed in Section 3.3.2 using an independent validation cohort.

#### 3.2.2. Gene-Level Comparative Analysis

Gene-level comparative analysis was conducted by mapping missense SNPs to their corresponding protein-coding genes and examining patterns of shared and breed-specific gene sets between populations. Pairwise comparisons revealed a substantial overlap in variant-associated genes between breeds. In the LES–SER comparison, 6184 genes were shared, while 1559 genes were unique to LES and 1385 genes were unique to SER. Similarly, the LES–THR comparison identified 6132 shared genes, along with 1611 genes specific to LES and 1374 genes specific to THR. In the SER–THR comparison, 6057 genes were common to both breeds, with 1509 genes unique to SER and 1446 genes unique to THR.

**Figure 6:**
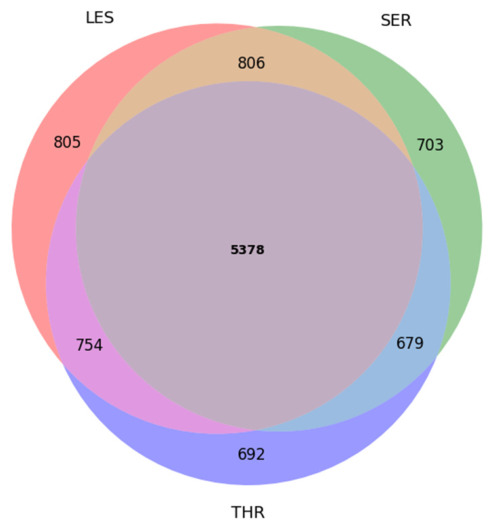
Weighted Venn diagram illustrating shared and breed-specific variant-associated genes among three Greek sheep breeds (LES, SER, THR).

To further characterize gene-level overlap across all populations, a three-way comparison was performed. The combined non-redundant gene catalogue comprised 9820 genes, of which 5378 were shared among all three breeds. Breed-exclusive gene sets included 805 genes in LES, 703 genes in SER, and 692 genes in THR, indicating that, despite substantial gene-level overlap, each breed retains a distinct subset of genes affected by coding variation. The results are illustrated in a Venn diagram (Figure 6).

#### 3.2.3. Functional Enrichment Analysis of Shared and Breed-Specific Gene Sets

Functional enrichment analysis was conducted on gene sets obtained from both pairwise and three-way comparisons of genes harboring missense SNPs to elucidate functional similarities and differences among breeds. Enrichment assessments were performed using Gene Ontology (GO) and KEGG pathway analyses, focusing on genes that are shared among breeds, as well as for those that are exclusive to specific breeds.

Across all pairwise comparisons (LES–SER, LES–THR, and SER–THR), the genes shared between breeds exhibited highly consistent functional enrichment profiles (Supplementary Table S2). Shared gene sets were enriched in biological processes related to cilium assembly and organization, microtubule-based cytoskeletal processes, cell projection assembly, and DNA damage response and repair. Enrichment in extracellular structure organization was additionally observed in the LES–SER and LES–THR comparisons, while cell cycle checkpoint–related processes were specifically enriched among genes shared between SER and THR. At the molecular function level, in all pairwise comparisons, shared gene sets were enriched in a consistent set of core terms, including regulation of small GTPase and nucleoside triphosphatase activity and nucleic acid–associated catalytic activity, often accompanied by ion channel or cation transport functions and lipid transport–related activity, depending on the breed pair (Supplementary Table S3). KEGG pathway analysis of pairwise shared gene sets revealed highly consistent enrichment patterns across all breed comparisons. In the LES–SER, LES–THR, and SER–THR comparisons, genes shared between breeds were predominantly enriched in pathways related to genome maintenance and DNA repair, cytoskeletal and muscle-related processes, and membrane transport and cell–matrix interaction. In the LES–THR comparison, enrichment additionally emphasized pathways associated with cytoskeletal organization in muscle cells and motor protein function, while the SER–THR comparison showed enrichment primarily in cytoskeletal/muscle-related and cell–matrix interaction pathways (Supplementary Table S4).

In contrast, breed-unique gene sets exhibited more heterogeneous enrichment patterns. Genes unique to LES were primarily associated with regulation of metabolic and biosynthetic processes, lipid metabolism, developmental and morphogenetic processes (including nervous system development), and cellular stress response. SER-specific genes showed enrichment in the regulation of RNA metabolism and gene expression, developmental and morphogenetic processes, metabolic and homeostatic regulation, organelle organization, and cellular stress response, including apoptosis-related processes. Genes unique to THR were enriched in developmental and morphogenetic processes, cytoskeletal organization and cellular architecture, metabolic and homeostatic regulation, organelle organization, cellular stress response, and immune-related processes (Supplementary Table S2). At the molecular function level, breed-exclusive gene sets exhibited more variable molecular function profiles. LES-specific genes were primarily associated with signaling regulation, including GTPase regulation, protein phosphorylation, and ubiquitination. SER-specific genes showed enrichment in transcriptional regulatory activity, oxidoreductase functions, chromatin-associated binding, and general catalytic and binding activities. Genes exclusive to THR were enriched in RNA processing and ATP-dependent RNA metabolic activity, metal ion transport, and general catalytic functions (Supplementary Table S3). However, no significant KEGG pathway enrichment was detected for breed-exclusive gene sets (Supplementary Table S4).

In the three-way comparative analysis, genes shared among LES, SER, and THR were consistently enriched in biological processes related to DNA damage response and repair, cilium assembly and organization, microtubule-based cytoskeletal processes, cell projection assembly, and cellular structural organization. At the molecular function level, the shared genes showed enrichment in ATP-dependent catalytic activity, nucleic acid–directed catalytic activity, nucleotide binding, enzyme regulatory activity, ion binding, lipid transporter activity, and regulation of small GTPase signaling. KEGG pathway analysis further identified enrichment in DNA damage repair pathways, cytoskeletal and extracellular matrix–based structural processes, and ABC transporter pathways. In contrast, breed-exclusive gene sets in the three-way comparison exhibited enrichment for developmental and morphogenetic processes, cellular stress response and survival, metabolic and homeostatic regulation, organelle organization, and immune-related processes. A summary of the enrichment results for the three-way shared and exclusive gene sets is presented in Table 4.

### 3.3. Validation of Candidate Missense SNPs Using MassARRAY Genotyping

To investigate whether the putatively breed-specific missense variants identified in the WGS analysis segregate at the population level, a targeted genotyping approach was employed. A panel of 46 missense SNPs was selected for validation and genotyped using the MassARRAY platform in an independent cohort of 54 animals, comprising 18 individuals from each of the three Greek indigenous sheep breeds (LES, SER, THR). Specifically, the panel included 30 SNPs identified as breed-specific in the discovery phase (10 per breed) and 16 SNPs detected in more than one breed in the WGS analysis (Supplementary Table S5). This validation step aimed at assessing the distribution of the candidate variants across breeds and at determining whether the patterns observed in WGS could be replicated at a population level.

#### 3.3.1. Genotyping Quality and Call Rates

Genotyping of the 46 candidate missense SNPs was successfully performed on a validation cohort of 54 animals using the MassARRAY platform. Genotyping quality was high across the dataset, with sample call rates ranging from 90% to 100%. The majority of samples exceeded 95% completeness (Supplementary Table S6). SNP-level coverage was also high, as most loci achieved 100% genotyping success, and all SNPs surpassed the predefined 90% call rate threshold (Supplementary Table S6). After quality control filtering, the final dataset included 46 SNPs genotyped across 54 individual animals, which were retained for subsequent allele frequency estimation and statistical analyses.

#### 3.3.2. Allele Frequency Distribution and Breed Differentiation

Allele frequencies for the validated SNP panel were calculated for each breed (LES, SER, and THR) using the MassARRAY genotyping dataset. Statistical comparisons of allele distributions among breeds were performed using Fisher’s exact tests, followed by Benjamini–Hochberg false discovery rate (FDR) correction.

Of the 46 validated SNPs, 17 exhibited statistically significant differences in allele frequencies between breeds after FDR correction (q < 0.05). These SNPs largely corresponded to variants initially classified as breed-specific in the WGS discovery phase. For example, several LES-associated variants, such as rs422734187 (*OR10C1*) and rs401664668 (*OR9A1P*), demonstrated substantially higher allele frequencies in LES compared to SER and THR. Similarly, SER-associated SNPs, including rs401183126 (*OR13A1*) and rs409873445 (*TRAV39*), showed elevated frequencies in the SER population, while THR-associated variants, such as rs410875969 (*OR2A12*) and rs594676125 (*OR1J4*), were most prevalent in THR animals. In addition to the statistically significant loci, eight SNPs followed the expected breed-specific allele frequency pattern, but did not reach statistical significance after multiple testing correction. These loci had the highest allele frequency in the predicted breed but exhibited more moderate differences between populations. Examples include rs413768160 (*KRTAP19-5*) in LES and rs421102706 (*CHD9NB*) in SER. A smaller subset of five SNPs displayed allele frequency distributions inconsistent with the patterns predicted by the WGS discovery analysis, indicating that these loci did not replicate the breed-specific pattern observed in the discovery dataset (rs399201339, 11:41098506, 2:229842947, rs1087597447). Finally, the 16 SNPs initially classified as common variants showed comparable allele frequencies between the three breeds and did not exhibit significant differentiation after FDR correction. Only one locus (rs412607607, *TRAV39*) displayed a more uneven allele distribution among breeds, although this difference was not statistically significant.

Detailed allele frequencies, statistical results, and classification of all validated SNPs are provided in Table 5.

## 4. Discussion

Indigenous sheep breeds represent an important reservoir of genetic diversity, shaped by long-term adaptation to local environments, management practices, and production systems [6]. A comprehensive understanding of the genomic architecture that underpins this diversity is crucial for the conservation of genetic resources, the improvement of breeding strategies, and the preservation of resilience in the face of environmental and climatic challenges [28]. Advances in whole genome sequencing have enabled the detailed characterization of genetic variation within livestock populations, thereby offering new opportunities for animal genomics through the identification of both conserved and population-specific genetic features.

In the present study, we investigated coding sequence variation across three Greek indigenous sheep breeds (Lesvos, Thrace, and Serres) to explore shared and breed-associated functional patterns within local sheep populations. To focus on variants with potential functional consequences, we prioritized protein-altering exonic variants, including missense SNPs and exonic indels, and examined their associated genes through GO and KEGG pathway enrichment analyses within both breed-level and comparative (pairwise and three-way) frameworks.

Overall, our analyses revealed a significant shared component of coding variation between breeds, along with distinct pools of breed-specific variants. Normalizing variant counts by gene length further identified genes with an increased polymorphism density within each breed, suggesting loci that may contribute to genetic differentiation among populations. Comparative functional enrichment consistently indicated a conserved functional backbone across breeds, primarily involving pathways related to genome maintenance, cilium and cytoskeletal organization, and core catalytic functions. At the same time, breed-associated functional signatures were evident, reflecting divergence within metabolic, structural, and regulatory processes.

Regarding breed-specific differences, among the three breeds examined, Lesvos (LES) exhibited a distinct functional profile, supporting previous studies showing genetic differentiation between mainland and island sheep in Greece [29]. This observation is consistent with the known production orientation of the LES breed, which is primarily dairy-oriented and has been reported to exhibit strong metabolic efficiency and adaptability to low-quality feeding conditions [14]. Unlike SER and THR, the LES breed is primarily confined to an island environment at the northeastern Greek–Turkish border, where geographic isolation and unique ecological conditions may have contributed to the accumulation of breed-associated genetic variation. The results of this study indicate that LES is characterized by enrichment in metabolic, biosynthetic, and sensory-related processes, as well as regulatory and transport-related molecular functions. These patterns were evident in both functional enrichment analyses of genes harboring prioritized missense SNPs and indels and in the identification of genes with elevated normalized polymorphism densities, as well as in the comparative analysis, suggesting coordinated variation within specific biological systems. Specifically, at the biological process level, LES showed enrichment in cofactor and metabolic processes, including pathways related to vitamin digestion and absorption, and specialized processes such as photoreceptor cell maintenance. These signals were further supported at the molecular function and KEGG pathway levels by enrichment in ABC transporter activity, glycosyltransferase and carbohydrate-modifying functions, and chromatin-associated binding and epigenetic regulation, indicating the potential involvement of nutrient handling, cellular transport, and regulatory control mechanisms. Such processes are particularly relevant in dairy breeds, such as LES [14], where nutrient absorption, transport, and metabolic regulation play a key role in milk synthesis and overall production efficiency. Together, these findings suggest a functional emphasis on metabolic flexibility and cellular regulation in the LES breed, which is consistent with its dairy production profile, although these associations should be interpreted with caution.

Genes exhibiting the highest SNP densities per kilobase in LES were strongly enriched for olfactory receptor (OR) genes (e.g., *OR10C1*, *OR1J4*, *OR9A1P*, *OR56A3*, *OR8I2*), as well as genes involved in immune and signaling functions (e.g., *TRAV39*) and those related to wool and skin structure (e.g., *KRTAP4-2*, *KRTAP36-1*, *KRTAP19-5*). Olfactory receptor gene families are known to tolerate high levels of sequence variation due to relaxed purifying selection and gene family expansion, and have been repeatedly implicated in environmental sensing, foraging behavior, and dietary adaptation in mammals [30,31]. The prominence of OR genes among highly polymorphic loci in LES suggests an increased standing variation in sensory perception pathways, which may reflect adaptation to heterogeneous grazing environments and diverse plant resources. The analysis of indel density in LES further highlighted genes associated with epidermal differentiation, barrier formation, and structural integrity. The highest indel frequencies were observed in genes such as *SPRR2A* and *SPRR4*, which encode small proline-rich proteins involved in keratinocyte differentiation and skin barrier assembly [32]. Similarly, *SCYGR6*, another gene associated with hair or wool structure, belongs to the Keratin-Associated Protein (KRTAP) Type 28 family. Furthermore, *KIAA0040*, identified in genomic studies, is linked to meat quality traits such as shear force (tenderness), where it is positively correlated with toughness and negatively with marbling/color scores, suggesting its role in muscle and fat development [33]. Notably, *OR10C1* also appeared among genes with elevated indel density, reinforcing the signal of extensive variation within sensory receptor loci. Together, these patterns suggest that genetic differentiation in LES may primarily reflect modulation of conserved metabolic, sensory, and structural systems, potentially supporting adaptation to localized grazing conditions and environmental heterogeneity.

SER, another breed examined in the present study, represents a mainland population that has been raised under less geographically constrained conditions than LES, with exposure to diverse environmental, nutritional, and management regimes. The results of this study revealed that SER is characterized by enrichment in structural, regulatory, and signaling-related processes, a pattern that is consistently observed in functional enrichment analyses of genes harboring prioritized missense SNPs and indels, as well as normalized polymorphism density analyses and comparative analyses. Such functional patterns may be consistent with the increased demands for tissue development, growth performance, and physiological regulation observed in dual-purpose breeds, such as SER [14]. At the biological process level, SER exhibited enrichment in genome maintenance and cell cycle regulation, cilium assembly and organization, microtubule-based cytoskeletal processes, and cell projection assembly, underscoring the significance of cellular architecture and regulatory control mechanisms. These findings were further supported at the molecular function level by enrichment in ion channel and calcium signaling activity, regulation of small GTPase and nucleoside-triphosphatase activity, nucleic acid–associated and ATP-dependent catalytic functions, along with lipid transport and lipid metabolic activity. These functions are directly related to energy balance and metabolic efficiency, which are essential for supporting both growth and lactation performance. KEGG pathway analysis corroborated this functional profile, revealing enrichment in DNA damage repair pathways, cytoskeletal and motor protein pathways, and ECM–receptor interactions, suggesting coordinated variation in pathways involved in cellular signaling, structural organization, and cell–matrix communication.

Analysis of normalized SNP density in SER identified a set of genes exhibiting elevated polymorphism, predominantly those involved in hair and skin structure, sensory perception, and immune-related signaling. Notably, *KRTAP4-2*, associated with wool and hair fibers [34], exhibited the highest SNP density, followed by several olfactory receptor genes (*OR13A1*, *OR4E1*, *OR6K6*, *OR10G2*, *OR6C68*, *OR56A3*), reinforcing the recurring observation of high variability within sensory receptor gene families. Additional polymorphic loci included *TRAV39*, linked to immune function and signaling [35], and *SCYGR4*, a gene identified in studies focusing on the genetic characterization of Anatolian sheep breeds [36]. A significant number of SNPs was also observed in *UCN2*, a highly expressed gene in skeletal muscle that regulates energy balance, glucose metabolism, and muscle mass [37], supporting its potential relevance to production traits such as growth performance and body composition. Genes exhibiting the highest indel densities in SER further highlighted variation in loci associated with structural integrity, metabolic processing, and regulatory control. The most prominent indel signal was observed in *KRTAP4-3*, followed by *CYP4F21*, *SPRR4*, *KIAA0040*, and *SCYGR6*. Keratin-associated proteins and small proline-rich proteins play key roles in epidermal differentiation and coat structure [32,34]. As mentioned above, *KIAA0040* is related to meat quality traits [33]. Furthermore, *CYP4F21* is a specific type of cytochrome P450 enzyme, first identified in sheep (ovine) seminal vesicles, functioning as a prostaglandin E2 20-hydroxylase, crucial for metabolizing prostaglandins and likely involved in reproductive processes [38,39]. Additionally, the indel-enriched gene *DSPP* encodes a crucial protein in tooth formation, with mutations leading to dentin defects [40]. Overall, the SER breed displays a pattern of genetic differentiation characterized by elevated variation in genes related to structural traits, sensory perception, immune signaling, and metabolic and regulatory functions, which may be consistent with fine-scale modulation of conserved biological systems in response to diverse mainland environmental and management conditions.

THR is another breed found in mainland Greece. Unlike the LES population, THR is raised in a continental region characterized by pronounced seasonal variation, mixed grazing systems, and diverse management practices—factors that may have shaped the observed breed-associated genomic signature. This is consistent with the characteristics of the THR breed, which is typically reared in extensive systems and is known for its resilience to challenging environmental conditions and seasonal variability [15]. Functional enrichment analyses of genes harboring prioritized missense SNPs in THR indicated enrichment in biological processes related to genome maintenance and DNA repair, cilium assembly and organization, microtubule-based cytoskeletal processes, cell projection assembly, and extracellular structure organization. Such processes may be particularly relevant in populations exposed to environmental stressors, where efficient cellular maintenance and repair mechanisms are essential. At the molecular function level, THR-specific enrichment included nucleic acid–associated and ATP-dependent catalytic activity, regulation of small GTPase and nucleoside-triphosphatase activity, ion channel activity, and functions related to lipid metabolism and ester hydrolysis. KEGG pathway analysis further supported this functional profile, with enrichment in genome maintenance and protein biosynthesis pathways, cytoskeletal and muscle-related pathways, ABC transporter pathways, ECM–receptor interactions, and metabolic pathways related to amino acid metabolism, including aminoacyl-tRNA biosynthesis and lysine degradation. These pathways are closely linked to metabolic adaptation and may support physiological responses to fluctuating nutritional availability.

Analysis of normalized SNP density in THR identified a set of genes with elevated polymorphism, primarily among loci involved in sensory perception, structural traits, and immune-related signaling. The highest SNP densities were observed in olfactory receptor genes, including *OR2A12*, *OR1J4*, *OR8I2*, *OR56A5*, *OR6C68*, *OR6N1*, *OR2T1*, and *OR13A1*, which may be consistent with adaptation to heterogeneous and seasonally variable grazing environments. Additional highly polymorphic loci included *KRTAP36-1*, associated with wool and hair fibers [34], *TRAV39* and *TRDV3*, linked to immune function and T-cell receptor signaling [35], which are important for maintaining health and resilience under extensive management conditions, and *UCN3*, a gene implicated in regulating stress responses, appetite, and glucose homeostasis [41,42], suggesting a potential role in physiological adaptation to environmental stress and resource variability. Together, these findings suggest enhanced standing variation in genes related to environmental sensing, structural traits, and immune responsiveness in THR. Genes exhibiting the highest indel densities in THR further emphasized variation in loci associated with metabolic processing, regulatory control, and cellular organization. The strongest indel signal was detected in *CYP4F21*, followed by *SCYGR6*, *OR10Q1*, *OR8I2*, and *OR6C68*. As previously discussed, *CYP4F21* encodes a cytochrome P450 enzyme involved in prostaglandin metabolism, suggesting potential relevance to reproductive and metabolic processes [38,39]. Additional indel-rich genes include *FOXL2*, a crucial transcription factor for ovarian development and function [43], and *BHLHE41*, recognized as a key transcriptional regulator with research highlighting its importance in immune cell function [44]. Also noteworthy are *CDK5R2* and *ZNF688*, which are implicated in transcriptional regulation, developmental processes, and cellular stress responses, consistent with the enrichment of regulatory and organelle-associated biological processes observed at the functional level. These findings indicate that THR has accumulated standing variation in genes associated with cellular architecture, metabolism, immune signaling, and environmental sensing, which may be consistent with adaptation to variable mainland environments and seasonal production conditions, although such interpretations should be considered with caution.

In addition to WGS analyses, population-level validation of prioritized missense SNPs provided further support for the robustness of the discovery-phase findings. Most loci showed allele frequency patterns consistent with the WGS-based classification, and several variants exhibited significant differentiation between breeds. These results suggest that many of the prioritized variants capture real population-level genetic differences rather than individual-specific variation. At the same time, a subset of loci did not replicate the expected pattern, which may reflect stochastic variation, allele frequency fluctuations in small populations, or the limited number of individuals included in the discovery phase. Such discrepancies are common in exploratory genomic studies and highlight the importance of validating candidate variants in broader population samples. Overall, the validation analysis strengthens confidence in the biological relevance of the identified loci and provides a basis for their potential use in future population genetic and breeding studies.

Analysis of the population-level distribution of candidate variants provides important insights into the genetic differentiation of breeds, even in the absence of direct genotype–phenotype associations. Several of the validated variants are located in genes with known biological functions relevant to economically important and adaptive traits. Specifically, variants within keratin-associated protein genes (KRTAP family) may be associated with wool and fiber characteristics, as these genes are key structural components influencing wool quality and fleece properties [45,46]. In addition, amino acid changes in KRTAPs have been shown to affect post-translational modifications and protein charge, potentially influencing fiber structure [47], while specific KRTAP variants have been associated with wool traits in different sheep breeds [48,49]. Similarly, *CDH15* is involved in muscle development and cellular proliferation [50] and has been reported to be differentially expressed in sheep with varying meat quality traits [51]. Genes such as *AURKA* and *ADAMTS3* have also been implicated in reproductive and developmental processes in sheep [52,53], suggesting potential relevance for production traits. Moreover, variants in *PRKDC*, a gene encoding the 460 kDa catalytic subunit of the DNA-dependent protein kinase, have been associated with growth-related traits in sheep [54], while *IL17B* may be associated with immune function and resilience, traits that are critical to animal health and adaptation to environmental stressors [55]. Notably, a substantial proportion of the validated variants are located in olfactory receptor (OR) genes (e.g., *OR10C1*, *OR1J4*, *OR9A1P*). Olfactory receptor gene families are known to exhibit high evolutionary turnover and variability between species and environments, reflecting adaptation to ecological niches. Recent evidence has shown that mammalian species, including sheep, adapted to specific environments, such as high-altitude habitats, exhibit a convergent reduction in functional olfactory receptor repertoires, suggesting that olfactory genes are subject to environment-driven selective pressures [56]. This supports the hypothesis that variation in OR genes may reflect ecological adaptation rather than direct production traits.

Although these observations do not establish direct causal relationships between specific variants and phenotypes, they provide biologically plausible links to traits of economic and adaptive importance. The observed population-level distribution of these variants represents a first step towards the identification of candidate markers that could be further explored in future genotype–phenotype association and functional studies. However, the direct functional effects of these specific variants on protein structure and function have not yet been experimentally validated. Therefore, these interpretations remain hypothesis-generating.

Building upon the breed-specific functional and gene-level signatures identified, the observed patterns of shared and unique coding variation among Greek indigenous sheep breeds carry important implications for conservation, traceability, and breeding strategies. While the conserved functional backbone among breeds highlights the necessity of preserving core genetic diversity essential for population fitness, the presence of distinct, breed-specific variant sets, particularly in genes related to sensory perception, productive traits, metabolism, and immune function, emphasizes the genetic uniqueness of each population. Genes and variants exhibiting increased polymorphism densities, particularly within olfactory receptor families, keratin-associated proteins, and regulatory loci, may represent informative genomic markers that could contribute to breed traceability and authentication, thereby supporting the protection of indigenous genetic resources. In addition, population-level validation of selected missense SNPs confirmed the expected allele frequency patterns for most loci, further supporting the relevance of the prioritized variants identified in the discovery phase. Concurrently, the functional annotation of these variants offers a valuable framework for future marker-assisted or genomic selection strategies that respect the breed identity while targeting traits pertinent to adaptation, resilience, and productivity in local environmental contexts. Collectively, these findings highlight the potential value of integrating genomic information into conservation and breeding programs aimed at safeguarding the long-term sustainability and adaptive capacity of Greek sheep breeds.

At this point, it is also important to compare our findings with the study by Tsoureki et al. (2025) [12]. While Tsoureki et al. (2025) [12] provided the first whole genome sequencing dataset for Greek sheep breeds and focused on variant discovery and dataset characterization, the present study extends this work by prioritizing functionally relevant coding variants and validating selected SNPs in an independent population, thereby providing additional biological and applied insights. Specifically, the novelty of our study lies in: (i) the targeted identification and prioritization of coding variants, with a particular focus on missense SNPs and indels with potential functional impact, (ii) the application of a dedicated bioinformatics filtering and comparative framework to identify breed-specific and shared variants, and (iii) the validation of selected candidate SNPs in an independent cohort of animals (*n* = 54; 18 per breed) using a MassARRAY-based genotyping approach.

Finally, several strengths and limitations of the present study should be acknowledged. A major strength is the focus on Greek autochthonous sheep breeds, which remain underrepresented in genomic and functional annotation studies compared to widely used commercial populations. To our knowledge, this study represents one of the first efforts to systematically characterize, functionally annotate, and compare SNP and indel variation across multiple indigenous Greek sheep breeds. An additional strength of the study is the population-level validation of prioritized missense SNPs using MassARRAY genotyping, which confirmed the allele frequency patterns of many candidate variants and provided further support for the biological relevance of the discovery-phase findings. Together, these analyses provide a valuable genomic resource for future conservation, traceability, and breeding initiatives. However, the relatively limited number of sequenced individuals constrains the resolution of individual-level variation and restricts the ability to detect robust selection signatures or establish direct genotype–phenotype associations. Nevertheless, the consistency of functional enrichment patterns observed across breed-level, pairwise, and three-way comparative analyses, together with population-level validation of prioritized SNPs, supports the biological relevance of the reported functional categories. A limitation of the present study is also the focus on coding variants, especially missense SNPs and indels, while regulatory regions (e.g., UTRs and upstream/downstream elements) were not explored. Although such variants may play an important role in gene expression and phenotypic variation, their interpretation remains complex. In addition, current genome annotations in livestock species are often based on widely studied or commercial populations, which may not fully capture regulatory variation in indigenous breeds.

Regarding future directions, future work should expand genomic analyses to include a broader range of indigenous Greek sheep breeds, providing a more comprehensive view of genetic diversity and adaptive variation. The present study focused on three representative breeds (Lesvos, Serres, and Thrace), selected based on sample availability and their geographical and phenotypic diversity. Specifically, recent sheep pox outbreaks in Greece have imposed significant limitations on sample collection, due to movement restrictions and culling of animals, including local breeds. In this context, documenting existing genomic variation is particularly important to support the conservation and sustainable management of these genetic resources. Future studies should also expand the analysis to include regulatory regions and integrate additional layers of genomic information (e.g., transcriptomic or epigenetic data), which would improve the functional interpretation of non-coding variants, particularly in underrepresented indigenous populations. Future studies could incorporate population genetic approaches, such as principal component analysis (PCA), admixture analysis, phylogenetic reconstruction, and tests of selection, to further investigate the evolutionary forces shaping genetic variation between breeds, too. Such analyses would provide deeper insights into population structure, gene flow, and potential signatures of selection, complementing the variant-centered approach applied in the present study. The inclusion of larger cohorts and genome-wide datasets will be essential for robust population-level inference, particularly in the context of indigenous breeds. Finally, future research should focus on the functional characterization of candidate variants using approaches such as protein structure modeling, in vitro assays, and integrative multi-omics analyses, which will provide deeper insights into their potential biological and phenotypic effects.

Overall, this study provides an initial framework for exploring the functional landscape of genomic variation in Greek sheep and underscores the need for future research that incorporates larger sample sizes, integrated phenotypic and environmental data, and expanded validation efforts to further elucidate the genetic basis of breed-specific traits and local adaptation.

## 5. Conclusions

In this study, we characterized and compared coding sequence variation across three indigenous Greek sheep breeds using whole genome sequencing, with a focus on protein-altering SNPs and exonic indels. Our analyses revealed a substantially conserved functional backbone across breeds, dominated by pathways related to genome maintenance, cytoskeletal organization, and core regulatory processes, along with suggestive breed-associated functional patterns. Integration of functional enrichment analyses with polymorphism density profiling highlighted genes and pathways potentially related to sensory perception, structural traits, metabolic regulation, and environmental response. To further evaluate the population-level relevance of candidate variants identified in the discovery phase, a subset of missense SNPs was validated in an independent cohort using MassARRAY genotyping. The majority of loci showed allele frequency patterns consistent with the WGS-based classification, with several variants displaying significant differentiation between breeds, while others followed the expected trends without reaching statistical significance. These results support the robustness of the variant prioritization approach and highlight loci that may contribute to genetic differentiation among the examined populations.

Together, these findings provide new insights into the functional landscape of genomic variation in Greek indigenous sheep breeds and establish a foundation for future studies aimed at exploring the links between genetic diversity and phenotypic traits, local adaptation, conservation, and sustainable breeding strategies. Overall, we emphasize that the functional interpretations presented in this study should be interpreted with caution and considered hypothesis-generating, as direct genotype–phenotype relationships and experimental validation are required to confirm these associations. In this context, while some observed patterns may be consistent with the distinct geographic origin and environmental conditions of the studied breeds, such associations should not be interpreted as direct evidence of adaptation, but rather as indicative of potential underlying biological processes that warrant further investigation.

## Figures and Tables

**Table 1:** Functional classification of SNPs identified in the initial variant dataset across the three studied breeds (LES, SER, THR). SNPs are categorized according to predicted functional impact at the protein level, including synonymous, nonsynonymous (missense), stop-gain, stop-loss, and variants with unknown annotation.

Breed/ Type of Mutation	LES	SER	THR
synonymous	48,739	46,849	47,298
nonsynonymous	32,593	31,422	31,520
stopgain	475	463	435
stoploss	32	29	34
unknown	18	20	25

**Table 2:** Top GO (BP, MF) and KEGG pathways enrichment results for genes harboring prioritized missense SNPs in the LES, SER, and THR sheep breeds.

Breed	Top Enriched BP Terms	Top Enriched MF Terms	Top Enriched KEGG Terms
LES	Genome maintenance and DNA repair processes, Cilium assembly and organization, Cofactor and metabolic processes, Cell–cell communication and conduction processes, Photoreceptor cell maintenance	Ion channel and calcium signaling activity, Regulation of vesicular trafficking and small GTPase signaling, Chromatin-associated binding and epigenetic regulation, Glycosyltransferase and carbohydrate-modifying activity, ABC transporter activity,	Genome maintenance and protein biosynthesis pathways, Cytoskeletal, motor, and muscle-related pathways, ABC Transporter pathways, ECM–receptor interaction, Vitamin digestion and absorption
SER	Genome maintenance and cell cycle regulation, Cilium assembly and organization, Microtubule-based cytoskeletal processes, Cell projection assembly	Ion channel and calcium signaling activity, Regulation of small GTPase and nucleoside-triphosphatase activity, Nucleic acid–associated and ATP-dependent catalytic activity, Lipid transport and lipid metabolic activity, Carbon–oxygen lyase activity	Genome maintenance and DNA repair pathways, Cytoskeletal (muscle cells) and motor protein pathways, ECM–receptor interaction
THR	Genome maintenance and DNA repair, Cilium assembly & organization, Microtubule-based cytoskeletal processes, Cell projection assembly, Extracellular structure organization	Nucleic acid–associated and ATP-dependent catalytic activity, Regulation of small GTPase and nucleoside triphosphatase activity, Ion channel activity, Lipid metabolic and ester hydrolysis activity	Genome maintenance and protein biosynthesis pathways, Metabolic and organelle-associated pathways (lysine degradation, peroxisome), Cytoskeletal and muscle-related pathways, ABC Transporter pathways, ECM–receptor interaction

**Table 3:** Top GO (BP, MF) enrichment results for genes harboring prioritized exonic indels in the LES, SER, and THR sheep breeds.

Breed	Top Enriched BP Terms	Top Enriched MF Terms
LES	Developmental & morphogenetic processes (anatomical structure, animal organ development, embryo development), Regulation of metabolic processes, Stress response, Organelle organization	Catalytic, hydrolase & transferase activities, Enzyme binding & regulation, Nucleoside phosphate binding, Ion binding
SER	Developmental & morphogenetic processes (anatomical structure, embryo development), Regulation of RNA metabolism and gene expression, Organelle organization, Stimulus & stress response	DNA binding, Catalytic, hydrolase & transferase activities, Enzyme binding & regulation, Nucleoside phosphate binding, Ion binding
THR	Developmental & morphogenetic processes (animal organ development), Regulation of RNA metabolism and gene expression, Stimulus & stress response, Organelle organization	Guanyl nucleotide exchange factor activity, Catalytic, hydrolase & transferase activities, Enzyme binding & regulation, Ion binding

**Table 4:** Top GO (BP, MF) and KEGG pathways enrichment results for genes harboring missense SNPs in the LES-SER-THR comparison.

Category	Top Enriched BP Terms	Top Enriched MF Terms	Top Enriched KEGG Terms
Shared	DNA Damage response & repair, Cilium assembly and organization, Microtubule-based cytoskeletal processes, Cell projection assembly, Cellular structural organization	ATP-dependent catalytic activity, Nucleic acid–directed catalytic activity, Nucleotide binding, Enzyme regulatory activity, Ion binding, Regulation of small GTPase signaling, Lipid transporter activity	DNA damage repair pathways (Homologous recombination), Cytoskeletal and ECM-based structural processes, ABC Transporter Pathways
Exclusive LES	Developmental and morphogenetic processes, Cellular stress response and survival, Metabolic and homeostatic regulation, Organelle organization and cellular architecture, Immune-related processes	Transferase activity, Ion binding, Enzyme binding, Chromatin binding, Hydrolase activity	NA
Exclusive SER			
Exclusive THR			

**Table 5:** Allele frequencies and statistical results for the 46 validated SNPs across the three sheep breeds in the validation cohort. The table includes gene annotation, WGS-based classification (breed-specific or common), allele frequencies (AF) in Lesvos (LES), Serres (SER), and Thrace (THR), and the FDR-adjusted q-values obtained from Fisher’s exact tests. The Pattern column indicates whether the allele distribution significantly supports the WGS classification (Significant), follows the expected trend without statistical significance (Consistent, NS), shows similar frequencies across breeds (Shared), or deviates from the expected pattern (Inconsistent).

SNP	Gene	WGS Classification	LES AF	SER AF	THR AF	*q*-Value	Pattern
rs422734187	*OR10C1*	LES-specific	0.72	0.17	0.24	1.0 × 10^−4^	Significant
rs3483482816	*OR1J4*	LES-specific	0.58	0.4	0.37	0.084	Consistent (NS)
rs401664668	*OR9A1P*	LES-specific	0.75	0.26	0.20	6.0 × 10^−5^	Significant
rs417884159	*KRTAP16-1*	LES-specific	0.6	0.22	0.25	1.7 × 10^−4^	Significant
rs423335178	*OR56A3*	LES-specific	0.65	0.28	0.33	2.1 × 10^−4^	Significant
rs408422273	*OR2T1*	LES-specific	0.62	0.34	0.30	3.3 × 10^−4^	Significant
rs399201339	*UQCRH*	LES-specific	0.46	0.50	0.48	0.74	Inconsistent
rs413768160	*KRTAP19-5*	LES-specific	0.61	0.35	0.32	0.1	Consistent (NS)
rs161752499	*ALDH4A1*	LES-specific	0.56	0.43	0.38	0.11	Consistent (NS)
rs589560256	*ZNF784*	LES-specific	0.69	0.21	0.28	1.2 × 10^−4^	Significant
11:41098506	*KRTAP4-2*	SER-specific	0.33	0.39	0.28	0.62	Inconsistent
rs401183126	*OR13A1*	SER-specific	0.23	0.73	0.21	5.8 × 10^−5^	Significant
7:2388825	*OR4E1*	SER-specific	0.30	0.67	0.34	1.3 × 10^−4^	Significant
2:229842947	*SCYGR4*	SER-specific	0.43	0.46	0.41	0.68	Inconsistent
rs421102706	*CHD9NB*	SER-specific	0.35	0.54	0.40	0.12	Consistent (NS)
rs428775201	*CTXND2*	SER-specific	0.32	0.5	0.37	0.14	Consistent (NS)
rs409873445	*TRAV39*	SER-specific	0.20	0.70	0.24	6.4 × 10^−5^	Significant
rs430501547	*SPZ1*	SER-specific	0.28	0.65	0.36	1.8 × 10^−4^	Significant
rs403620280	*B3GALT9*	SER-specific	0.37	0.52	0.38	0.13	Consistent (NS)
rs405518297	*OR10J5*	SER-specific	0.27	0.62	0.29	1.6 × 10^−4^	Significant
rs410875969	*OR2A12*	THR-specific	0.22	0.26	0.69	6.0 × 10^−5^	Significant
rs594676125	*OR1J4*	THR-specific	0.19	0.31	0.66	9.7 × 10^−5^	Significant
13:42782435	*UCN3*	THR-specific	0.23	0.31	0.74	8.5 × 10^−5^	Significant
rs430282808	*KRTAP24-1*	THR-specific	0.36	0.44	0.54	0.090	Consistent (NS)
rs419510057	*TRDV3*	THR-specific	0.28	0.36	0.61	3.1 × 10^−4^	Significant
rs160159964	*HRCT1*	THR-specific	0.30	0.38	0.59	4.2 × 10^−4^	Significant
12:62604417	*ZNF648*	THR-specific	0.26	0.30	0.63	1.8 × 10^−4^	Significant
rs1087597447	*RPL36AL*	THR-specific	0.45	0.44	0.47	0.61	Inconsistent
rs597823465	*LENEP*	THR-specific	0.39	0.43	0.52	0.11	Consistent (NS)
rs430184133	*KIAA0319*	THR-specific	0.24	0.34	0.64	1.4 × 10^−4^	Significant
rs413413850	*PATJ*	Common	0.41	0.52	0.46	0.32	Shared
2:526519	*OR13J1*	Common	0.73	0.66	0.70	0.36	Shared
rs193636766	*LDLR*	Common	0.19	0.27	0.22	0.41	Shared
rs405049897	*OR10G6*	Common	0.58	0.49	0.54	0.27	Shared
rs430446886	*SLC44A4*	Common	0.31	0.39	0.34	0.46	Shared
rs421315179	*CDH15*	Common	0.68	0.61	0.65	0.34	Shared
rs419387521	*KIAA1217*	Common	0.44	0.38	0.41	0.48	Shared
rs413198514	*ZNF169*	Common	0.24	0.36	0.29	0.44	Shared
rs595830546	*KLHL33*	Common	0.14	0.21	0.18	0.47	Shared
rs405666320	*FAM204A*	Common	0.79	0.71	0.75	0.40	Shared
rs415183610	*AURKA*	Common	0.48	0.56	0.51	0.45	Shared
rs412607607	*TRAV39*	Common	0.52	0.29	0.48	0.09	Shared
rs160646919	*PRKDC*	Common	0.61	0.69	0.64	0.39	Shared
rs409400253	*ADAMTS3*	Common	0.08	0.16	0.11	0.43	Shared
3:164283039	*OR6C68*	Common	0.69	0.73	0.66	0.37	Shared
rs421900588	*IL17B*	Common	0.54	0.63	0.57	0.38	Shared

## Data Availability

The raw WGS data are openly available at NCBI’s Sequence Read Archive, under BioProject ID PRJNA1246525. The genotyping data are available within the manuscript and its supplementary materials.

## Supplementary Materials

The following supporting information can be downloaded at: https://www.mdpi.com/article/10.3390/cimb48050480/s1.

## Abbreviations

The following abbreviations are used in this manuscript:

BP	Biological Process
GO	Gene Ontology
LES	Lesvos breed
MF	Molecular Function
SER	Serres breed
SNP	Single-Nucleotide Polymorphism
THR	Thrace breed
WGS	Whole Genome Sequencing
