# Supplementary material for: Comparative Whole Genome Analysis and Targeted Validation of Variants in Three Greek Indigenous Sheep Breeds
Source: Curr Issues Mol Biol. 2026 May 5;48(5):480. doi: 10.3390/cimb48050480 (PMC13204572; doi:10.3390/cimb48050480)
Supplement: Supplementary file 1 [file cimb-48-00480-s001.zip › Supplementary Tables S2-S4.pdf]

***Supplementary Table S2.*** *Top GO BP enrichment results for genes harboring missense SNPs in pairwise comparisons of the three breeds (LES, SER, THR).*

| **Comparison Level** | **Category** | **Top Terms** |
|---|---|---|
| **Pairwise LES–SER** | Shared LES–SER | Cilium assembly & organization,<br>Microtubule-based processes,<br>Cell projection assembly,<br>DNA damage response & repair,<br>Extracellular structure organization |
| | Unique LES | Regulation of metabolic and biosynthetic processes,<br>Developmental and regulatory processes (nervous system development),<br>Cellular stress response,<br>Immune-related processes |
| | Unique SER | Regulation of RNA metabolism and gene expression,<br>Developmental and morphogenetic processes,<br>Cellular stress response and survival-related processes (apoptosis),<br>Immune-related processes |
| **Pairwise LES–THR** | Shared LES–THR | Cilium assembly & organization,<br>Microtubule-based processes,<br>Cell projection assembly,<br>DNA damage response & repair,<br>Extracellular structure organization |
| | Unique LES | Lipid metabolic processes,<br>Developmental and morphogenetic processes (nervous system development),<br>Cellular stress response |
| | Unique THR | Developmental and morphogenetic processes,<br>Cytoskeletal organization and cellular architecture, |

| Comparison Level | Category | Top Terms |
|---|---|---|
| | | Immune-related processes |
| Pairwise SER–THR | Shared SER–THR | Cilium assembly & organization,<br>Microtubule cytoskeleton organization,<br>Cell projection assembly,<br>DNA damage response & repair,<br>Cell cycle checkpoint signaling |
| | Unique SER | Developmental and morphogenetic processes,<br>Metabolic and homeostatic regulation,<br>Organelle organization,<br>Cellular stress response and survival-related processes (apoptosis),<br>Immune-related processes |
| | Unique THR | Developmental and morphogenetic processes,<br>Metabolic and homeostatic regulation,<br>Organelle organization,<br>Cellular stress response,<br>Immune-related processes |

***Supplementary Table S3**. Top GO MF enrichment results for genes harboring missense SNPs in pairwise comparisons of the three breeds (LES, SER, THR).*

| Comparison Level | Category | Top Terms |
|---|---|---|
| Pairwise LES–SER | Shared LES–SER | Ion channel and calcium signaling activity,<br>Regulation of small GTPase and nucleoside-triphosphatase activity,<br>Nucleic acid–associated catalytic activity,<br>Glycosyltransferase and carbohydrate-modifying activity |
| | Unique LES | Regulation of small GTPase signaling,<br>Protein ubiquitination and post-translational modification, |

| Comparison Level | Category | Top Terms |
|---|---|---|
| | | General catalytic activity,<br>Molecular binding functions (ion binding, carbohydrate derivative binding) |
| | Unique SER | Oxidoreductase and dioxygenase activity,<br>Transcriptional regulatory activity,<br>General catalytic activity,<br>Molecular binding functions (ion binding, carbohydrate derivative binding) |
| **Pairwise LES–THR** | Shared LES–THR | Ion channel and cation transport activity,<br>Regulation of small GTPase and nucleoside-triphosphatase activity,<br>Nucleic acid–associated catalytic activity,<br>Lipid transport and lipid metabolic activity |
| | Unique LES | Regulation of small GTPase and nucleoside-triphosphatase activity,<br>Protein phosphorylation and kinase activity |
| | Unique THR | General catalytic activity (transferase, hydrolase activity)<br>Ion binding |
| **Pairwise SER–THR** | Shared SER–THR | Cytoskeletal binding and structural interaction,<br>Regulation of small GTPase and nucleoside-triphosphatase activity,<br>Nucleic acid–associated catalytic activity,<br>Lipid transport and lipid metabolic activity |
| | Unique SER | General catalytic activity (transferase, hydrolase activity),<br>Molecular binding and protein interaction (ion and enzyme binding),<br>Chromatin-associated binding and regulation |

| Comparison Level | Category | Top Terms |
|---|---|---|
| | Unique THR | RNA processing and ATP-dependent RNA metabolic activity,<br>Protein ubiquitination and post-translational modification,<br>Metal ion transmembrane transport activity |

***Supplementary Table S4.*** *Top KEGG pathways enrichment results for genes harboring missense SNPs in pairwise comparisons of the three breeds (LES, SER, THR).*

| Comparison Level | Category | Top Terms |
|---|---|---|
| **Pairwise LES–SER** | Shared LES–SER | Genome maintenance and DNA repair pathways,<br>Cytoskeletal and muscle-related pathways,<br>Membrane transport and cell–matrix interaction pathways |
| | Unique LES | NA |
| | Unique SER | |
| **Pairwise LES–THR** | Shared LES–THR | Genome maintenance and DNA repair pathways,<br>Cytoskeletal (muscle cells) and motor protein pathways,<br>Membrane transport and cell–matrix interaction pathways |
| | Unique LES | NA |
| | Unique THR | |
| **Pairwise SER–THR** | Shared SER–THR | Genome maintenance and DNA repair pathways,<br>Cytoskeletal and muscle-related pathways,<br>Cell–matrix interaction pathways |
| | Unique SER | NA |
| | Unique THR | |
